# Corneal complications following Post Kala-azar Dermal Leishmaniasis treatment

**DOI:** 10.1371/journal.pntd.0006781

**Published:** 2018-09-17

**Authors:** Shomik Maruf, Proggananda Nath, Muhammad Rafiqul Islam, Fatima Aktar, Azim Anuwarul, Dinesh Mondal, Ariful Basher

**Affiliations:** 1 Nutrition and Clinical Services Division (NCSD), International Centre for Diarrhoeal Disease Research, Bangladesh (icddr,b), Dhaka, Bangladesh; 2 Infectious and Tropical Medicine Department, Mymensingh Medical College and Hospital (MMCH), Mymensingh, Bangladesh; 3 Department of Ophthalmology, Mymensingh Medical College and Hospital (MMCH), Mymensingh, Bangladesh; Addis Ababa University, ETHIOPIA

## Abstract

Post Kala-azar Dermal Leishmaniasis (PKDL) is a sequel of Visceral Leishmaniasis (VL). The patients act as a reservoir for the causative parasite (i.e. *Leishmania donovani*) and thus should be diagnosed and treated with the utmost urgency to prevent the transmission of the disease. In this study, we tried to report the first instances of corneal complications supposedly associated with Miltefosine (MF), in PKDL patients and the probable pathophysiology of such events. The recently rejuvenated National Kala-azar Elimination Program in Bangladesh has put great emphasis on monitoring all the leishmaniasis patients to investigate possible adverse drug reactions (ADR). A total of 194 patients have received Miltefosine for the treatment of Post Kala-azar Dermal Leishmaniasis. So far five patients were found to have developed unilateral ophthalmic complications during the periods from May 2016 to October 2017, after being treated with MF for PKDL. Unfortunately, one of whom had to go through complete evisceration of the affected eyeball. Despite the fact that MF is the only oral formulation of choice to treat PKDL, occurrences of such unexpected ADRs after MF administration urges the exploration of the pathogenesis of such incidents and determine measures to avert such occurrences from happening in future.

## Author summary

PKDL is a sequel of VL, which acts as a source of leishmaniasis and should be diagnosed and treated at the earliest possible time to prevent disease transmission. In Bangladesh during the period from May 2016 to October 2017, a total of 5 patients were diagnosed to have Miltefosine induced unilateral ophthalmic complications. Miltefosine was introduced as an anti-parasitic drug through drug repurposing, and it has a complex mechanism of acting towards the parasite killing. The mechanism itself in certain circumstances may produce ophthalmic complications by its metabolites or by causing dry eye disease. Miltefosine is the only oral drug for leishmaniasis. Hence the etiopathogenesis and prevention strategy of such severe adverse events must be explored.

## Introduction

Post Kala-azar Dermal Leishmaniasis (PKDL) is a skin manifestation which appears as a macular, papular, or nodular rash or a combination of the three, usually starting from the face but may start from any other part of the body, typically after being treated for VL [1, 2]. Bangladesh is now in the consolidation phase for VL elimination after reaching the target of less than 1 cases /10000 population in all the endemic sub districts. As a result policymakers in Bangladesh are now keen to sustain this achievement. As PKDL cases can act as a source of infection, its early diagnosis and treatment is now a major concern [3]. Currently, Miltefosine (MF), amphotericin B deoxycholate, and liposomal amphotericin B (LAmB) are recommended by the World Health Organization as treatment options for PKDL [4]. MF or hexadecylphosphocholine (HePC, C21H46NO4P) is an amphiphilic cationic phospholipid analogue comprising of two parts, a hydrophobic chain of 16 carbons and a polar head group of phosphocholine [5,6]. The drug was initially developed as an anti-tumor agent, particularly for the treatment of cutaneous metastases of breast carcinoma, however, it is more commonly used as an anti-leishmanial drug, and preferably to treat PKDL cases [7]. As an immunomodulator, MF stimulates T-cells, macrophages and the expression of interleukin 3 (IL-3), granulocyte-macrophage colony stimulating factor (GM-CSF), and interferon gamma (INF-gamma) [8].

**Fig 1:**
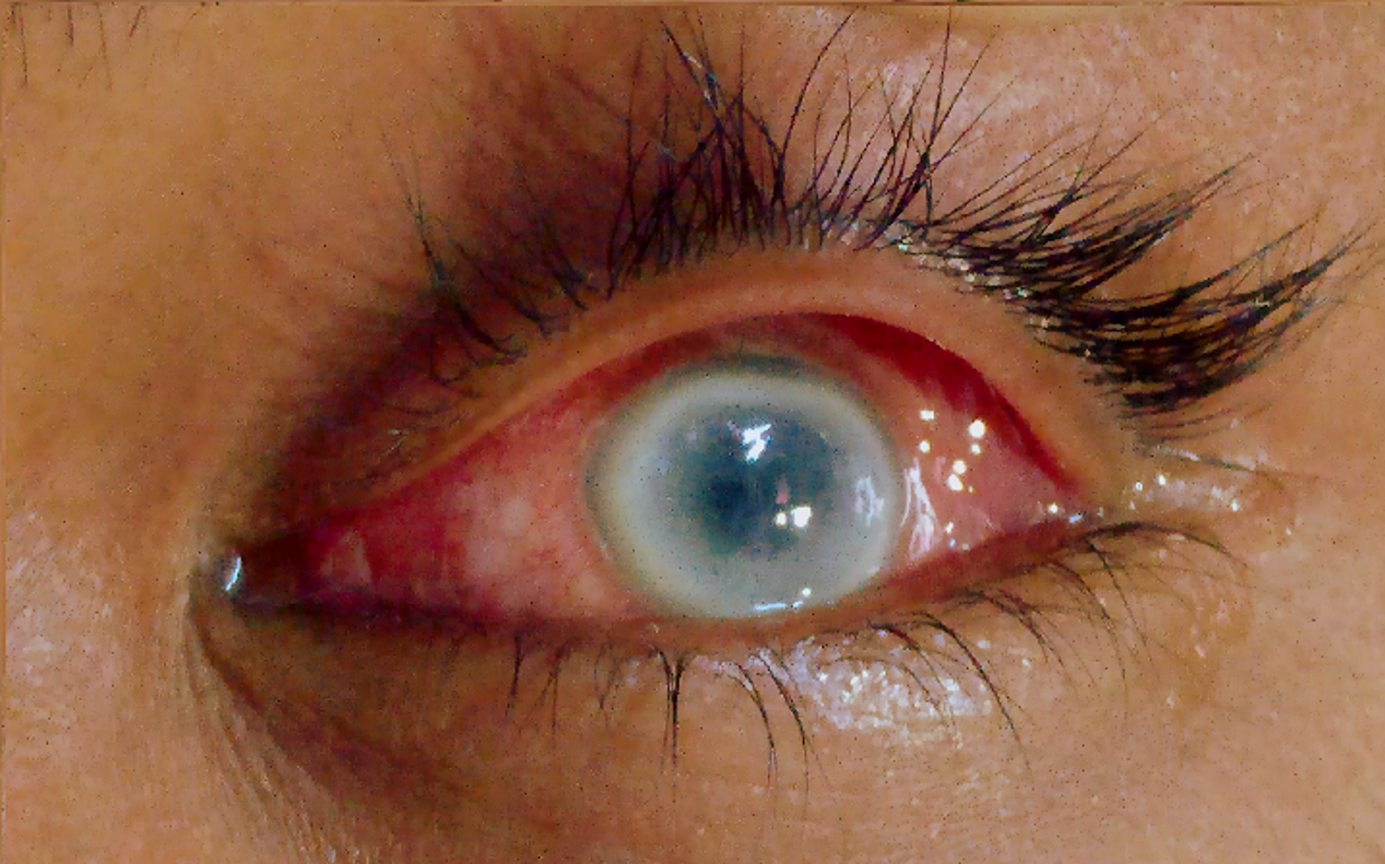
**Marginal keratitis (left)**. Showing 360° Peripheral corneal infiltration with ulceration with congested conjunctiva.

This report presents five cases of PKDL with lesions of various degrees who were prescribed with Capsule Miltefosine (50mg) twice daily for 84 days. All the patients developed unusual ophthalmic complications, after a varying duration of oral MF intake *(Fig 1)*. The drug is categorized as H319, by GHS Hazard Statement and there are reports of retinal toxicity in some animal studies. [9,10]. However, to the best of our knowledge, such severe adverse event (SAE) manifesting corneal toxicity with suspected association with MF, either in animal or human study, are the first instances in Bangladesh so far.

## Methods

The cases were referred to Surya Kanta Kala-azar Research Center (SKKRC)—a tertiary referral center for leishmania cases, for the diagnosis and treatment of PKDL between the periods of May 2016 to October 2017. A total number of 194 PKDL cases were treated during this period and 5 patients experienced such SAEs. For this study, we used hospital records to collect retrospective hospital data of the cases, but did not obtain any ethical approval. SKKRC is a research facility and the hospital takes a written consent from all the patients to use their hospital records. Furthermore, all the data were used after receiving written permission from the in-charge (medical officer and Head of the facility) of SKKRC and anonymity of all the patients were ensured.

The following is our effort to explain such strange serious adverse event in relation to our findings. All of the cases were immuno-competent and none were suffering from any other concomitant diseases during the treatment period, e.g. diabetes, hypertension. However, drug levels (pharmacokinetics) of the patients could not be assessed as they received treatment from the hospital outdoors and were at their home until the complication occurred. The individual details of the patients are given in *Table 1*.

**Table 1:** Case summary.

Parameter	Case 1	Case 2	Case 3	Case 4	Case 5
Age (years)	60	18	16	15	34
Address (District)	Jamalpur	Mymensingh	Mymensingh	Mymensingh	Mymensingh
Sex	Male	Male	Male	Male	Male
BMI (kg/m^2^)	19.1	20.4	18.7	21.2	20.8
Diagnosis (LD detection)	Microscopy & qPCR	Microscopy	Microscopy & qPCR	Microscopy & qPCR	qPCR
Type of lesion	Macular	Maculo-nodular	Macular	Maculo-nodular	Maculo-papular
**Treatment History for VL**					
Medication	MF 50 mg twice daily for 28 days	Single Dose LAmB	MF 50 mg twice daily for 28 days	Single Dose LAmB	SSG
Duration since Rx (years)	11	04	07	2.5	08
**Description of PKDL**					
Location	Generalized	Generalized	Generalized	Generalized	Generalized
Duration (Months)	60	24	12	07	60
Primary/ relapse	Primary	Primary	Primary	Primary	Relapse (2^nd^)
Prior Rx for PKDL	NA	NA	NA	NA	Multi-dose LAmB
**Treatment for PKDL**					
Rx prescribed	Cap. MF 50mg—twice daily for 84 days	Cap. MF 50mg—twice daily for 84 days	Cap. MF 50mg—twice daily for 84 days	Cap. MF 50mg—twice daily for 84 days	Cap. MF 50mg—twice daily for 84 days
Medication (Trade Name)	Miltefos^1^	Miltefos^1^	Impavido^2^	Miltefos^3^	Miltefos^3^
medication received	33 days	30 days	84 days	60 days	20 days
**Development of SAE**					
Complaints	a, b, c (left), d, e	a, c (left)	a, c (left), e, f	a, b, c (right), e, f	a, b, c (left)
Days of MF intake before symptom development	33	30	40	45	20
**Ophthalmic Examination**					
Visual Acuity	3/60	4/60	1/60	6/30	3/60
Congestion of Conjunctiva	Present	Present	Present	Present	Present
Corneal Infiltration	360° Peripheral	Peripheral–Up to 2mm tissue from limbus involving all layers of cornea.	Peripheral infiltration extending towards the center with presence of hypopyon	Peripheral infiltration extending towards the center with presence of hypopyon	Peripheral
Corneal Ulceration	Present	Present	Present	Present	Absent
Involved Eye	Left	Left	Left	Right	Left

a = painful eye, b = increased lacrimation, c = redness of eye, d = photophobia, e = dimness of vision, f = white spot, MF = Miltefosine, LAmB = Liposomal Amphotericin B, SSG = Sodium Stibogluconate

^1^ Cap. Miltefosine 50 mg (Popular Pharmaceuticals Ltd., Batch No.–SG J02)

^2^ Cap. Miltefosine 50 mg (Endo Venturex Limited, Batch No.– 2K5312A)

^3^ Cap. Miltefosine 50 mg (Popular Pharmaceuticals Ltd., Batch No.–SLL 21)

### Case summary

#### Case 1

A 60-year-old male from Jamalpur district was admitted in Surja Kanta Kala-azar Research Center (SKKRC), Mymensingh with the complaints of progressive non-itching hypomelanotic macular lesions for the last five years. He had a previous history of Visceral Leishmaniasis (VL) 11 years back and was treated with Cap. Miltefosine (50mg) twice daily for 28 days. His general and systemic examinations revealed no other abnormalities. The skin sensation was intact on the affected sites. Both microscopic examination and qPCR of the skin following a positive rK39 test confirmed the presence of LD body and DNA respectively. The patient was then prescribed to take Cap. Miltefosine (Miltefos, Popular Pharmaceuticals Ltd., Batch No.–SGJ02), 100 mg, in two divided doses for 84 days as per national guideline. After taking the drug for 33 days the patient experienced mild pain, increased lacrimation and redness of the left eye followed by photophobia and marked dimness of vision. He was advised to stop the medicine as soon as he contacted SKKRC and was referred to Department of Ophthalmology, Mymensingh Medical College and Hospital (MMCH), where they diagnosed him as a case of Mooren’s ulcer on the basis of visual acuity 3/60, matted eyelashes, congested conjunctiva and 360° peripheral corneal infiltration with ulceration. He was treated with topical antibiotics, steroid and lubricant eye drops. He was eventually treated with multi-dose LAmB without facing any adverse events, after his eye condition went back to normal. On 6 month after treatment follow up, his skin qPCR for LD-DNA was negative and there was no ophthalmic complaint.

#### Case 2

An 18-year-old male from Fulbaria, Mymensingh district came to SKKRC with progressive maculo-nodular lesions for the last two years. He gave a history of suffering from VL 4 years back for which he was treated with single Dose LAmB. In the beginning, there were only macular lesions on the face and arms, but later nodular lesions started to appear on his chin, cheek, lip, tongue and both hands, feet, and buttock. General examinations revealed no abnormality except the skin lesions over the above-mentioned sites. No hepato-splenomegaly was found and the lesions were non-itching with intact sensitivity. His diagnosis was confirmed through rK39 strip test followed by microscopic examination of skin biopsy confirming the presence of LD bodies. He was also treated with Cap. Miltefosine (Cap. Miltefos, Popular Pharmaceuticals Ltd., Batch No.–SGJ02) following the same protocol as case 1. About a month after having Cap. Miltefosine the patient developed pain and red coloration of the left eye. At first, he went to the local Upazilla health complex (secondary health care facility at the Sub-district level in Bangladesh) where the doctors treated him as a case of conjunctivitis but the condition was not improved and was eventually referred to SKKRC. The doctors at SKKRC stopped his medication and referred him to department of ophthalmology, MMCH. His ophthalmic examination revealed visual acuity—4/60, photophobia, congested conjunctiva, peripheral corneal infiltration extending up to 2 mm of corneal tissue from limbus, involving all the layers of the cornea and he was diagnosed as Marginal keratitis (left). He received specific treatment under the supervision of National Institute of Ophthalmology & Hospital (NIO&H), Dhaka. As soon as he felt better, he was treated with multi-dose LAmB at SKKRC. On 6 month after treatment follow up, his skin qPCR for LD-DNA was negative and there was no ophthalmic complaint.

#### Case 3

A 16-year-old male from Bhaluka, Mymensingh was admitted to SKKRC with non-itching macular skin lesions all over the body for a year. He had a history of VL seven years back and was treated with Cap. Miltefosine (50mg) twice daily for 28 days and was cured. On examination, the patient was non-febrile, non-anaemic and no hepato-splenomegaly was found. Laboratory investigations revealed he was rK39 positive and LD bodies were found under direct microscopy of skin biopsy. His qPCR for LD-DNA of skin biopsy was also positive. After confirming the diagnosis he was treated as a PKDL case with Cap. Miltefosine, (50mg), (Cap. Impavido, Endo Venturex Limited, Batch No.– 2K5312A) two capsules daily for 84 days. About one and a half month after medication he developed painful, red eye and dimness of the vision along with a white spot in the left eye. He was initially treated by village doctor and continued to take Miltefosine. As the condition worsened, he went to a local eye consultant who diagnosed him as a case of corneal ulcer and treated accordingly. Despite having no progress regarding his complication, he did not contact SKKRC and came for his scheduled follow up after completing the full dose of Miltefosine. The physician at SKKRC immediately referred him to MMCH ophthalmology department for further management. After examination, they found visual acuity 1/60, congested conjunctiva, and peripheral corneal infiltration with ulceration which extends towards the center and presence of hypopyon and was diagnosed as a case of Peripheral ulcerative Keratitis with secondary bacterial infection. Unfortunately it was too late for a recovery and doctors had to go for the evisceration of the eye with baseball implantation. He was further approached to take treatment for PKDL with LAmB but he refused to take any treatment.

#### Case 4

**A** 15-year-old male from Gafargaon, Mymensingh was admitted to SKKRC with non-itching papulo-macular skin lesions all over the body for 7 months. He had a history of VL 2.5 years back and was treated with single dose LAmB and got cured. On examination, the patient was non-febrile, not- anemic and no hepato-splenomegaly was found. His rK39 RDT was positive and direct microscopy of skin biopsy showed LD bodies. qPCR for LD-DNA was also positive. After confirming the diagnosis he was treated as a PKDL case with Cap. Miltefosine, (50mg), (Cap. Miltefos, Popular Pharmaceuticals Ltd., Batch No.–SLL 21) two capsules daily for 84 days. About 7 weeks later he developed painful, red eye with watery discharge and dimness of vision along with marginal white discoloration of the left eye. Despite the instructions, he didn’t stop taking the medicines for another two weeks and then contacted the physicians at SKKRC. The physician at SKKRC immediately stopped his medication and referred him to ophthalmology department, MMCH for further management. After examination, they found visual acuity 3/60, congested conjunctiva, and peripheral corneal infiltration which extends towards the center and presence of hypopyon and was diagnosed as a case of Marginal Keratitis. He was treated with topical antibiotics in addition to steroid and lubricant eye drops. He was administered LAmB at SKKRC after recovery from eye complications. On 6 month after treatment follow up, his skin qPCR for LD-DNA was negative and there was no further ophthalmic complaints.

#### Case 5

34-year-old male from Gafargaon, Mymensingh was admitted to SKKRC with non-itching nodulo-macular skin lesions all over the body for 5 years, with a history of VL 8 years back and was treated with SSG. He was previously treated twice for PKDL by multi-dose AmBisome, but his condition had not improved. On examination, the patient was non-febrile, slightly anemic and no hepato-splenomegaly was found. Laboratory investigations revealed he was rK39 positive and LD-DNA was found by qPCR of skin biopsy. After confirming the diagnosis he was treated as a PKDL case with Cap. Miltefosine, (50mg), (Cap. Miltefos, Popular Pharmaceuticals Ltd., Batch No.–SLL 21) two capsules daily for 84 days. About 3 weeks later he developed a painful, red eye with watery discharge. He immediately contacted physicians at SKKRC and was advised to stop taking the medication and was given steroid eye drops. After a week he recovered from his complications and was treated with multidose LAmB at SKKRC without further complication. On 6 month after treatment follow up, his skin qPCR for LD-DNA was negative and he had no similar ophthalmic complaint.

#### Treatment approach

All the cases were treated as a case of conjunctivitis initially by the ophthalmologists, as none could relate the possibility of the events to leishmaniasis or its treatment. As there was no improvement in the condition, eventually they went to SKKRC, where they were asked to stop taking MF. They were initially treated under the supervision of the Department of Ophthalmology, Mymensingh Medical College Hospital (MMCH) with topical antibiotic, steroid and lubricant eye drops and followed up each week until recovery. While all the other cases had recovered from the ophthalmic complications within two weeks, unfortunately, Case 3 required the evisceration of the eye with baseball implantation. After recovery from the ADRs, all the patients except case-3 were treated for PKDL multi-dose LAmB (20 mg/ kg body-weight of LAmB in four equally divided doses). No adverse events occurred while treating with this treatment regimen. The treated patients were followed up at 6 months after treatment and skin biopsies were collected for qPCR to detect LD-DNA, which yielded negative results.

## Results/Discussion

The findings gave some interesting insights, as all five cases were male having unilateral eye involvement (left eye in 4 out of 5 cases) and complications intensified mostly after the 5th week since the start of the treatment. Also, adolescents were more severely affected than the adults (Case 3 and 4). Male PKDL patients having a higher exposure to MF compared to female PKDL patients (AE Kip et al, Ph.D. thesis), might explain males being at a higher risk to develop such SAE. However, a retrospective clinical data is not sufficient to draw any conclusion regarding these curiosities.

Drug-induced corneal keratopathy is usually asymptomatic, often dose-related and may reflect the potential risk for lenticular or retinal changes, though may not always require cessation of therapy. However, with drugs also known to cause retinal or optic nerve toxicity, regular observation, and testing are necessary to reduce the risk of visual acuity loss [11].

The corneal changes are often the result of the underlying chemical properties of medications. Drugs having both amphiphilic and cationic properties, tend to produce a vortex pattern of deposits because of the accumulation of phospholipids [11]. This might be associated with drug-induced phospholipidosis or other mechanisms [12]. These symptoms typically resolve following drug cessation if done early, as we observed in four cases. The exception was case 3, who continued with his treatment in spite of the complications, resulting in permanent damage to his left eye.

MF is metabolized mainly by phospholipase D, releasing choline, choline-containing metabolites, and hexadecanol, which are likely to enter the intermediary metabolism and is almost completely eliminated. The drug keeps accumulating until the end of treatment due to long elimination half-lives [5]. The cationic amphiphilic properties of the drugs lead to intra-lysosomal accumulation of lipids. This stimulates two theories: the first is that, specific lysosomal phospholipases which normally would be responsible for catabolizing the lipids are inhibited [13]. The second is that the cationic amphiphilic properties allow the drugs to form drug-lipid complexes to enter the lysosomes, where it gets trapped after being protonated in the lysosomal acidic environment and become unable to pass through the hydrophobic bilayer surrounding the lysosome [13]. The trapped drug then binds with polar lipids to form lipid drug complexes which are resistant to digestion by phospholipases [13]. The tendency towards recovery after drug cessation might be explained as a consequence of unstable complexes developing increased susceptibility to phospholipases [14].

Another possible explanation is pre-existing or drug-induced dry eye disease (DED)—an autoimmune disorder characterized by inflammation of the ocular surface structures, causing burning, and itchy eyes that may lead to corneal ulceration, reduced vision, and even in some cases to blindness [15]. The etiology is unknown and somehow controversial but it is thought to be related to the release of pro-inflammatory cytokines in inflamed ocular structures. The effect of auto-reactive CD4+T helper cells, expressing the Th1 cytokines. Interleukin-2 and interferon-γ, amplified by MF induced Interleukin-3 secretion. Th1, Th2, and Th17 cells, as well as B cells, play important roles in aggravating DED [16]. Insufficient tear production or increased tear evaporation due to decreased lipid production from the disturbed function of meibomian glands can provoke DED [16].

Furthermore, the skin around the eyelids and the cornea are parasitized by leishmania [17]. Miltefosine may penetrate to these sites and induce parasite killing. Local immune responses, could also play an important role in this regard. This phenomenon may be unique to Miltefosine, owing to its capability to penetrate the skin.

Several studies have tried to explain the various consequences of VL in human including ophthalmic complications, such as retinopathy, keratitis, uveitis etc. A study in Sudan by Khalil, et al. 2011, demonstrates the occurrence of pan-uveitis as a consequence of VL either with or without the presence of PKDL lesions. There, one patient suffered ophthalmic complication even when she was not under ongoing treatment. They suggested that systemic spreading and infiltration of the *leishmania* spp and their accompanying inflammation might contribute to this phenomenon. However, in that study, the patients’ ophthalmic complications were managed by Sodium Stibogluconate (SSG) along with steroid eye drops [18, 19]. Another study in India reported keratitis in PKDL patients receiving treatment with MF. They were unable to find any offending growth in the detailed microbiological workup and suggested it could happen as a result of the immune response in cornea due to antigens released by the dying parasites [20].

Whereas, in our study, all the patients had long history VL (2.5–11 years) and PKDL (07–60 months) and they suffered from ophthalmic complications only after administering Miltefosine. All the four patients who had recovered, stopped taking Miltefosine after developing complications and received treatment only with steroid and lubricant eye drop and topical antibiotics. They were treated with LAmB only after their ophthalmic complications subsided and no further adverse events were observed. This indicates a possibility that Miltefosine may have an association to the complications.

The study, however, had several limitations. The data was collected retrospectively and the facilities for a more detailed investigation and examination were scarce at the study site. Only a small number of patients could be examined as this is a rare phenomenon. However, this situation warrants a prospective observational study of the patients receiving Miltefosine to establish this SAE.

### Conclusion

As if now, Miltefosine (MF) is the only available oral preparation for treating leishmaniasis patients. However, such SAEs certainly requires further exploration regarding its safety, more specifically when used for a longer duration. An effective pharmacovigilance activity can play a pivotal role in this regard. Despite, the scarcity of the evidence, physicians must meticulously monitor patients while MF is administered and ensure periodic follow-ups. Preemptive counseling of the patients concerning its side effects will enable them to identify complications in due time and seek appropriate help. The drug brochure should specify and elaborate these effects, so that the physicians can be aware of the possible adverse effects and coordinate with the primary care physician for consideration of dose reduction or alternative choice of therapy in the events of such complications.
